# Medicinal Plant Extracts

**DOI:** 10.3390/plants10050838

**Published:** 2021-04-22

**Authors:** Laura Grațiela Vicaș, Mariana Eugenia Mureșan

**Affiliations:** Faculty of Medicine and Pharmacy, University of Oradea, 1 Decembrie Street, 410073 Oradea, Romania; marianamur2002@yahoo.com

The therapeutic benefits of medicinal plants are well known and have been collected as important data on ethnomedicine [1]. This data can be a starting point in the use of medicinal plant species, whose use as alternative medicine should be based on scientific assessment, via chemical, biological, and pharmacological evaluation [2,3].

The study of medicinal and aromatic plants identified by the authors will be carried out according to a selection of the most used species, based on the country or area where the research is being conducted [4]. As such, in this special edition, the authors will be able to demonstrate their scientific contributions through the botanical description of medicinal plants—both traditionally and currently used species—as well as the chemical characterization of their respective phytocomplexes and their proven pharmacological action [5,6].

Results will be presented in a scientific manner with the intention of providing a source of reference for specialists, i.e., pharmacists, doctors, biologists, chemists, and other members of the scientific community [7,8]. The published articles will provide information based on scientific data obtained from ethno-pharmaceutical studies, experiments carried out through research projects, and the analysis of information available in the scientific literature [9].

The initial impetus for the creation of this special volume arose from the desire to provide correct information about the application of classical and innovative extraction methods, but also the scientific application of obtained extracts in the treatment of specific diseases. It is important that the authors also take into consideration the various states of conservation of the plant species, which can be achieved by analyzing their monographic data. The authors will be able to bring to light different species of medicinal and aromatic plants, some of them widely used in traditional and modern medicine, others less known or used. The large-scale use of plant supplements has enabled us to observe the current population’s increased interest in aspects of overall health and wellness. This perspective has determined us to bring to the attention of interested specialists the results regarding the most efficient extraction methods, the characterization of phytocomplexes, their demonstrated pharmacological activity, and their possible side/adverse effects.

Starting with the premise that any study commences with an in vitro system and is ultimately confirmed by an in vivo system, it could be stated that the toxicity and efficacy of the therapeutic and pharmacologically active substances can thus be more accurately assessed. Consequently, cell culture studies are simple methods used to study cellular processes or to test a therapeutic compound [10,11]. Experimental studies based on cell cultures are useful to research the effects of bioactive compounds extracted from medicinal plants. Moreover, the reproducibility of these methods permits them to be frequently used to test molecular mechanisms or cytotoxicity.

For this issue, relying on scientific data, we aim to ensure the durable valorization of the selected medicinal plant species, as well as their entry into alternative medicine.
